# Effect of Specific Oil Surface Area on the Thermal Stressing of Rapeseed Oil During Heating in an Electric Frying Pan

**DOI:** 10.1007/s11746-015-2770-9

**Published:** 2016-01-09

**Authors:** Jakub P. Kobyliński, Krzysztof Krygier, György Karlovits, Aleksandra Szydłowska-Czerniak

**Affiliations:** Faculty of Food Sciences, Warsaw University of Life Sciences, ul. Nowoursynowska 159, 02-776 Warsaw, Poland; Bunge Europe Research and Development Center, ul. Niepodległości 42, 88-150 Kruszwica, Poland; Faculty of Chemistry, Nicolaus Copernicus University in Toruń, 7 Gagarin Street, 87-100 Toruń, Poland

**Keywords:** Rapeseed oil quality, Pan heating, Specific oil surface, Oil height

## Abstract

The effect of specific oil surface (SOS) during pan frying of rapeseed oil on its thermal stability and antioxidant capacity (AC) was evaluated. Rapeseed oils with different oil layer heights (OLH = 0.5, 1.0, 1.5, 2.0, and 2.5 cm) were heated on an electric frying pan coated with Teflon at 180 ± 10 °C until a selected end point of 25 % total polar compounds (TPC) was reached. The changes of chemical parameters of oil samples such as peroxide value, *p*-anisidine value, Totox value, free fatty acids, TPC and AC using the 2,2-diphenyl-1-picrylhydrazyl assay were determined. Irrespective of the applied methods, the highest changes in oil with OLH = 0.5 cm were observed. Heating in low OLH also led to the fastest time of TPC formation in rapeseed oil; the 0.5-cm layer reached 25 % TPC in a relatively short time (71.5 min) compared to the highest OLH = 2.5 cm (*t* = 315.1 min). The SOS and the rate of change in the heated oils decreased with increasing OLH. Crucial effects of SOS on physicochemical oil changes were observed. The present study demonstrated the protective effect of increasing the OLH on the quality of the heated rapeseed oils.

## Introduction

Frying is one of the oldest methods of food preparation. It improves the sensory quality of food by the formation of aroma compounds, attractive color, crust and texture, but a large number of negative reactions take place [1]. Chemical reaction rates during frying depend on: characteristics of the frying medium and fried product, real concentration of unsaturated fatty acids, concentration of oxygen on the oil surface and in the oil, temperature and presence of catalyst heavy metals and surface active components [2]. Those reactions such as: hydrolysis, oxidation, isomerization, polymerization and cyclization give rise to a lot of desirable and non-desirable compounds [3]. The main difference between deep frying and pan frying is the amount of oil—less oil is used in the pan frying process and resulting in a higher value of the specific oil surface (SOS) [1]. As indicated by Soheili *et al*. [4] pan frying appeared to be a very deteriorative process. Moreover, a much higher levels of toxic lipid oxidation products during shallow frying than deep frying under the same conditions were determined [5]. This may be a result of different values of SOS—the ratio of the surface in contact with air on the weight of oil, which is significantly higher during pan frying. The frying stability of oil is inversely proportional to the SOS in the fryer. Under domestic cooking conditions the surface of the oil in contact with the air is often constant (when someone always uses the same pan). Also the mass of oil, which means in practice—the height of oil is the pan is the main factor influencing the SOS value [6].

The determination of total polar compounds (TPC) has been recognized worldwide as the most reliable method to assess oil deterioration during heating or frying. TPC include polar substances such as monoacylglycerols, diacylglycerols and free fatty acids (FFA), which occur in unheated oils, as well as polar transformation products formed during heating or frying of food. A limit for rejection of 25 % TPC in frying fats have been established in Poland [7].

Deterioration of fat may be slower in the presence of antioxidants. They act as free radical scavengers and quenchers of the formation of singlet oxygen [8]. Tocopherols and phenolic compounds such as phenolic acids (mainly sinapic acid and its derivatives in rapeseed oil), monohydroxy and dihydroxy phenols, flavonoids are of great importance as natural antioxidants of vegetable oils. Also carotenoids, phytosterols, phytostanols and phospholipids can improve oil stability during the frying process. The effect of frying on antioxidant capacity (AC) of vegetable oils was estimated. A significant decrease in DPPH values after frying of various edible oils was observed (DPPH = 22.0–87.0 % and DPPH = 3.2–68.0 % for the unheated and heated oils, respectively) [3, 8].

However, very limited research has been carried out on pan frying. Changes in the AC, oxidative stability, fatty acid composition, peroxide values (PV) and amounts of FFA, TPC, conjugated dienes, trienes, unsaturated aldehydes, ketones, polyphenols, phytosterols, tocopherols, squalene, carotenoids, chlorophylls in the non-fortified and fortified vegetable oils before and after pan frying were studied [2, 9–11].

However, there has been no reference to the determination of oxidative degradation and the AC of rapeseed oil after pan frying. Furthermore, to the best of our knowledge, no work has been reported on the relationship between oil layer height (OLH), SOS and the time of its deterioration.

The aim of the presented paper was to evaluate the effect of SOS during heating of fully refined rapeseed oil on an electric frying pan at temperature (180 ± 10 °C) on its thermal stability and antioxidant potential. Deterioration of the heated oils was monitored by determination of their PV, *p*-anisidine (*p*-AV), Totox values, FFA, TPC amounts. Also, the AC of the unheated and heated rapeseed oils was determined by the DPPH method. Moreover, correlations between the content of primary and secondary oxidation products and the AC of the studied rapeseed oil samples were examined and discussed.

## Experimental Procedures

### Reagents

All reagents were of analytical or HPLC grade. Acetic acid (100 %), isooctane, potassium iodide, sodium thiosulfate standard solution, potassium iodate volumetric standard, hydrochloric acid, ethyl alcohol, sodium hydroxide and phenolphthalein were purchased from Merck (Warsaw, Poland). 2,2-Diphenyl-1-picrylhydrazyl radical (DPPH, 95 %), (±)-6-hydroxy-2,5,7,8-tetramethyl-chromane-2-carboxylic acid (Trolox—TE, 97 %) and *p*-anisidine (99 %) were supplied by Sigma-Aldrich (Steinheim, Germany). Methanol (99.8 %) and *n*-hexane (95 %) were obtained from Avantor (Gliwice, Poland). CAPSens test oils for calibration were from C-Cit Sensors AG (Wädenswil, Switzerland). Redistilled water was used for preparation of solutions.

## Materials

Fully refined rapeseed oil (RO) in the original packing (poly(ethylene terephthalate) was kindly donated by ZT Kruszwica, (Bunge, Poland). It was stored at 4 °C until used in the frying.

### Heating Equipment and Experimental Conditions

A round electric frying pan (42.5 cm in diameter, surface area of the pan, *S* = 1418 cm^2^) with a non-stick TEFLON^®^ coating and with a glass lid was used to heat the oil samples under typical domestic frying conditions at 180 ± 10 °C. The pan had a total electrical power of 1500 W and the possibility to regulate it at 5 heating levels. (Ellrona Party, Caso International GmbH, Arnsberg, Deutschland). The oil temperature was measured with a thermometer (model SAF-T-LOG with penetration temperature probe 300 mm, model K, ETI Ltd Easting Close, Worthing, West Sussex). Temperatures were monitored continuously to ensure isothermal treatment at 180 °C for all samples. Changing the level of oil in pan—five different SOS (1.0, 1.3, 1.7, 2.5 and 5.1 cm^2^/g) were created. The SOS was calculated from the equation: SOS = *S*/*m* (cm^2^/g), where *S* is the surface of oil in contact with atmospheric oxygen (cm^2^) and *m* is the mass of oil (g). Because the surface area of the pan [*S* (cm^2^)] was constant, in further discussion the simplified term “oil layer height” (OLH) is also used.

Every 10 min, the oil was mixed and 1 mL of oil was taken from the surface to determine the TPC. The end of the heating period was when the TPC reached 25 % in the heated oil. Aliquots of the heated oils (200 g) were taken at the end of the experiment and stored at −18 °C until analysis. All of the heating experiments were performed in duplicate.

### Chemical Analyses

The PV of the studied oil samples was measured according to the potentiometric end-point determination method explained by ISO Method 27107: 2010 (E) [12] with use of Titrando 905 from Methron. The *p*-AV was analyzed as a measure of secondary oxidation products in the oil according to ISO Method 6885:2006 [13]. After determination of PV and *p*-AV, Totox values (TV) were calculated according to the formula (TV = 2PV + *p*-AV), which was proposed by ISO Method 6885: 2006 [13]. Free fatty acids (FFA) were measured according to the ISO Method 660:1996 and expressed as oleic acid [14].

The production of polar compounds in frying oil was assessed by determination of the TPC. This degradation of oil during frying was analyzed using the calibrated Frying Oil Sensor (FOS) CapSens 5000 instrument (C-CIT AG, Switzerland). Each sample was measured after cooling it to *T* < 120 °C. The oils’ frying life (time to reach 25 % TPC) was calculated from linear equations: *y* = (0.0554 ± 0.0036)*x* + (7.6020 ± 0.0132), *y* = (0.0696 ± 0.0036)*x* + (10.2230 ± 0.0014), *y* = (0.1113 ± 0.0030)*x* + (5.3743 ± 0.3031), *y* = (0.1805 ± 0.0095)*x* + (2.8156 ± 0.2483), *y* = (0.3524 ± 0.0327)*x* + (1.8181 ± 0.0569) plotting three times for each OLH = 2.5, 2.0, 1.5, 1.0 and 0.5 cm, respectively.

The AC of rapeseed oils before and after pan frying was determined according to the procedure described by Szydłowska-Czerniak *et al*. [15] and expressed as micromoles of Trolox equivalents per 100 g of the oil samples. In brief, 0.5 mL of methanolic extracts of oils was added to 1.5 mL of methanol and 0.5 mL of DPPH methanolic solution (304.0 μmol L^−1^). The mixtures were shaken vigorously and left in darkness for 15 min. The absorbance was measured at 517 nm against a reagent blank (2 mL of methanol + 0.5 mL of DPPH methanolic solution) using a Hitachi U-2900 spectrophotometer (Tokyo, Japan) in a 1-cm quartz cell. Five calibration curves were plotted by the least-squares method resulting in equation: %DPPH = (688.6 ± 8.1)c_TE_—(1.1 ± 0.5), *R*^*2*^ = 0.9991 and relative standard deviation of slope (RSD_slope_) = 1.89 %.

### Statistical Analysis

The PV, *p*-AV, FFA and TPC in the studied rapeseed oils were determined in triplicate, whereas the AC of five portions of each oil extract was analyzed within 1 day by the DPPH method for each of two heating sessions. The results obtained were presented as: mean c ± standard deviation (SD). One-way analysis of variance (ANOVA), followed by the Duncan test, was performed to analyze the significant differences between data (*p* < 0.05). Moreover, the Pearson correlation test was applied to determine the correlations between thermal stability and the AC of rapeseed oil samples. Differences of *p* < 0.05 were considered significant. StatGraphics Plus 5.0 and Excel were used for analyzing data and elaborating oil frying life based on linear equations.

## Results and Discussion

### Chemical Quality of Unheated Rapeseed Oil

The unheated refined rapeseed oil revealed initial values of PV = 0.09 mequiv O_2_/kg, *p*-AV = 1.2, Totox = 1.4 and FFA = 0.10 %, respectively (Table 1).

**Table 1:** Frying conditions and chemical parameters of the unheated and heated rapeseed oils with different layer heights

Rapeseed oil samples	Frying conditions						Chemical parameters			
	OLH (cm)	TOV (mL)	TOM (g)	SOS (cm^2^/g)	PV* ± SD (mequiv O_2_/kg)	*p*-AV* ± SD	Totox	FFA* ± SD (%)	t (25 % TPC)* ± SD (min)	DPPH^#^ ± SD (μmol TE/100 g)
RO unheated	–	–	–	–	0.09 ± 0.00^a^	1.2 ± 0.06^a^	1.4^a^	0.10 ± 0.00^a^	–	459.5 ± 19.6^f^
RO1	0.5	300	279	5.1	45.15 ± 2.34^e^	235.4 ± 1.7^e^	325.7^e^	0.29 ± 0.01^e^	71.5 ± 3.9^a^	3.3 ± 0.1^a^
RO2	1.0	600	558	2.5	23.35 ± 0.10^d^	214.2 ± 2.2^d^	260.9^d^	0.27 ± 0.01^d^	123.2 ± 7.8^b^	22.2 ± 0.9^b^
RO3	1.5	900	837	1.7	17.80 ± 0.36^c,d^	202.6 ± 2.0^c^	238.2^c^	0.24 ± 0.00^c^	176.5 ± 2.1^c^	46.9 ± 1.2^c^
RO4	2.0	1200	1116	1.3	14.85 ± 0.37^b,c^	190.9 ± 2.8^b^	220.6^b^	0.23 ± 0.01^b^	212.5 ± 4.5^d^	60.5 ± 1.8^d^
RO5	2.5	1500	1395	1.0	9.50 ± 0.65^b^	192.4 ± 0.6^b^	211.4^b^	0.24 ± 0.01^b,c^	315.1 ± 20.8^e^	72.6 ± 1.9^e^

Values are means ± standard deviations

Different letters (a–f) within the same column indicate significant differences between analytical parameters of the refined rapeseed oils before and after heating with different layer heights (one-way ANOVA and Duncan test, *p* < 0.05)

*OLH* oil layer height in pan, *TOV* total oil volume, *TOM* total oil mass, *SOS* specific oil surface, *t* (*25* *% TPC*) time to achieve 25 % TPC

^a^ *n* = 6 (3 × 2 heating sessions), ^b^ *n* = 10 (5 × 2 heating sessions); *p* = 0.05

These quality factors were within the desirable level for fresh frying oil (PV < 5 mequiv O_2_/kg, *p*-AV < 8, Totox < 10, FFA < 0.15 %) [16]. However significantly higher PV values (0.75–14.0 mequiv O_2_/kg) for different fresh vegetable oils were reported by other authors [2, 3, 8, 17–19]. Although, these edible oils before heat treatment revealed similar *p*-AV values (0.00–3.67) and FFA content (0.04–3.33 %), whereas Totox values were higher (2.8–8.6) [2, 3, 17–19].

Moreover, the content of polar compounds (6.6 %) in oil before frying was significantly lower than the prescribed limit of TPC of 25 % [7]. However, the unheated rapeseed oil contained a higher amount of TPC when compared to TPC = 0.9–6.0 % in various fresh oils determined by Andrikopoulos *et al*. [2], Kalantzakis *et al*. [3], Karakaya and Şimşek [8] and Sebastian *et al*. [17].

Also the AC results listed in Table 1 suggest that the rapeseed oil before pan frying had the highest DPPH value (459.5 μmol TE/100 g). For comparison, radical scavenging activity, expressed as % reduction in concentration of DPPH was the highest for unheated vegetable oils (22.0–87.0 %) [3, 8].

### Oxidative Changes of Rapeseed Oils

Irrespective of the oil layer height (OLH), PV, *p*-AV and Totox increased significantly (*p* < 0.05) after heating (Table 1). In the smallest OLH = 0.5 cm the fastest increase of those quality factors was observed. This was expected considering that during heating of a thin oil layer (as in the case of pan frying) there is a greater oxygen absorption per unit oil than during heating in a larger amount of oil (deep frying). These results suggest a significant increase in the rate of oxidation and reduction in the frying life of the oil [4]. In addition, the Duncan test indicated that the heated rapeseed oils (depth in pan 2.0 and 2.5 cm) did not differ significantly in PV, *p*-AV and Totox values (Table 1). Moreover, sample RO3 (OLH = 1.5 cm) had a similar amount of hydroperoxides as samples RO2 (OLH = 1.0 cm) and RO4 (OLH = 2.0 cm).

The considerable formation of *p*-AV confirmed the decomposition of primary oxidation products (hydroperoxides) to the secondary ones (alcohols, ketones, aldehydes and acids). It is noteworthy that, *p*-AV values of the studied rapeseed oils ranged between 1.2 and 235.4 and differed significantly (Duncan test, Table 1).

With increasing OLH, lower amounts of oil oxidation products were formed—the total degree of oxidation of an oil expressed as Totox was significantly low in the case of OLH = 2.0 and 2.5 cm. As can be seen the Totox value increases above 150-fold after heating rapeseed oil in OLH = 2.0 and 2.5 cm, while 230-fold for OLH = 0.5 cm. The obtained results of PV, *p*-AV and Totox values indicate that the negative effect of SOS on the oil frying life may be reduced with increasing the OLH in the frying equipment. Although, PV (9.50–45.15 mequiv O_2_/kg), *p*-AV (190.9–235.4) and Totox (211.4–325.7) values of all heated oil samples exceeded the maximum acceptable levels, which are 5, 8 and 10 mequiv O_2_/kg, respectively.

For comparison, PV, *p*-AV and Totox results for different vegetable oils after deep and pan frying as well as microwave treatment ranged between 2.85 and 247.5 mequiv O_2_/kg, 2.50–56.5 and 14.3–516.2 [2, 8, 17–19].

It can be noted that the calculated values of RSD = 0.00–6.84 %, 0.31–5.00 %, respectively, indicate reasonable repeatability of the standard methods for PV and *p*-AV determinations.

### Free Fatty Acids Formation in Rapeseed Oils

It is evident that FFA amounts in all oil samples after pan frying is approximately 2.5 times higher than in fresh rapeseed oil (Table 1). The highest content of FFA (0.29 %) was observed in the case of the smallest OLH = 0.5 cm and with increased OLH lower amounts of FFA were formed. However, the Duncan test indicated that RO3, RO4 and RO5 samples (OLH = 1.5, 2.0 and 2.5 cm, respectively) did not differ significantly in the level of FFA (Table 1). If food was fried, more FFA were formed. This is due to emission of water and steam from food, which accelerates the rate of triacylglycerol decomposition and causes the formation of components like free fatty acids [17]. Therefore, FFA content in various oils from the fryer after deep frying of different food products prepared in restaurants and pan frying of potatoes varied widely between 0.07 and 4.30 % [2, 17, 19].

It can be noted that the applied official method for FFA analysis had a good precision, expressed as RSD = 0.00–4.35 %.

### Total Polar Compounds in the Heated Rapeseed Oils

Formation of polar compounds, which indicates oil deterioration, is strongly related with the primary and secondary oxidation that takes place during frying [8]. There are no worldwide regulations and guidelines for the control of frying fats, but a number of European countries have promulgated specific laws and regulations concerning limits for TPC for used frying oils. For the purpose of this test, we chose the limit for TPC as 25 % for determination of frying life of used oils, according to the Polish regulations [7].

**Fig. 1:**
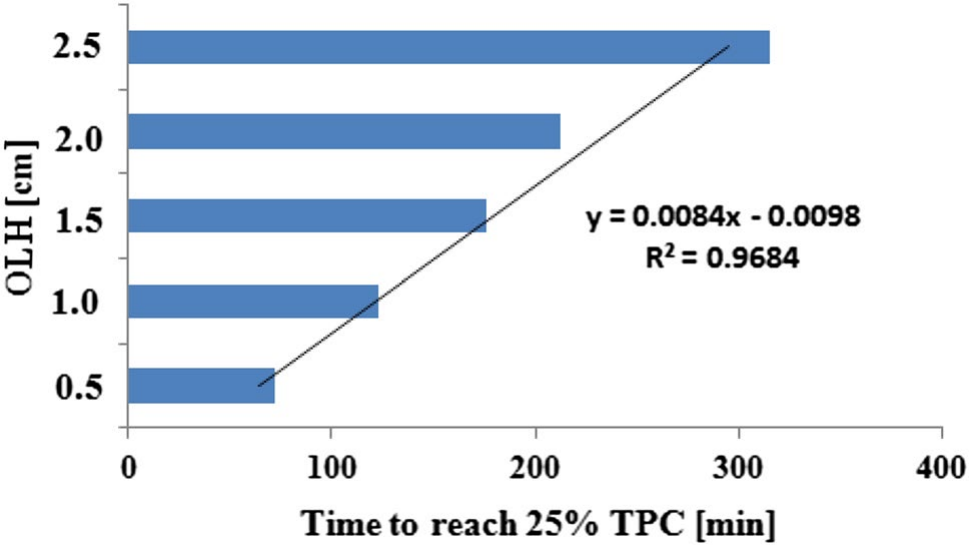
Time to reach 25 % total polar compounds in rapeseed oils depending on the oil layer height

The results of TPC accumulation as a function of time are presented in Table 1 and Fig. 1.

It can be seen that heating time of rapeseed oils to reach 25 % TPC at constant temperature progressed linearly (*y* = 0.0084*x* − 0.0098, *R*^*2*^ = 0.9684) as the OLH increased (Fig. 1).

The results indicated that each rapeseed oil sample significantly differs from others (*p* < 0.05) in time to reach 25 % TPC (Duncan test, Table 1). The fastest rate of TPC accumulation was observed in the thinner layer of oil. With increasing OLH, time to reach 25 % TPC in the studied rapeseed oils lengthened (Table 1). It is noteworthy that, the average time to reach 25 % TPC in RO5 (OLH = 2.5 cm) was about 4.5 times longer than for sample RO1 (OLH = 0.5 cm). This is probably due to SOS, which has the highest value (5.1 cm^2^/g) in 0.5 cm oil level (Table 1). However, Andrikopoulos *et al*. [2], Kalantzakis *et al*. [3], Karakaya and Şimşek [8] found lower concentrations of TPC in different vegetable oils after deep frying (2.77–18.5 %) for 25–300 min and pan frying (17.4–19.3 %) for 60 min. In addition, the TPC levels were lower in frying oils during using (4–15.5 %) and discarded frying oils (6.5–15.0 %) at selected restaurants (except one, TPC = 37.5 %) [17]. Only similar TPC amounts (23.5–29.9 %) in virgin and refined olive oils, sunflower, soybean cottonseed oils, as well as a commercial blend after 600 min frying were determined by Kalantzakis *et al*. [3].

The RSD results (1.19–6.60 %) indicate the method used was precise for the determination of TPC in the heated rapeseed oils.

### Antioxidant Capacity of Rapeseed Oils

It is noteworthy that fresh rapeseed oil had the highest antioxidant capacity (DPPH = 459.5 μmol TE/100 g), whereas the obtained DPPH results of the heated rapeseed oils ranged between 3.3 and 72.6 μmol TE/100 g (Table 1). Therefore, heating of rapeseed oil with different layer heights resulted in a large decrease in the AC (84.2–99.3 %). This fact can be explained as that natural components such as tocopherols, some sterols and phospholipids with antioxidant activity in oil are lost during the heating process [3, 8, 10]. As presented in Karakaya and Şimşek study [8], phenolics may not be active compounds but tocopherols may be responsible for slowing oxidation of the oils.

It is evident from Table 1 that the DPPH results for each of the studied rapeseed oils are significantly different from one another (Duncan test, *p* < 0.05). The highest percentage loss (99.3 %) of the AC was observed for the heated sample RO1 (OLH = 0.5 cm). Increasing of the OLH resulted in lower losses of oil AC. Although rapeseed oils with higher OLH were heated longer than RO1 sample (OLH = 0.5 cm), they revealed higher DPPH values at the end of the process (Table 1). This fact indicates the protective effect of SOS reduction. For comparison in the report of Kalantzakis *et al*. the fresh edible oils (DPPH = 28.4–78.9 %) gradually lost their radical-scavenging activity after 2.5 h (DPPH = 9.7–68.6 %), 5 h (3.8–56.0 %), 7.5 h (3.6–40.9 %) and 10 h (3.2–40.2 %) of heating at 180 °C [3]. A similar effect of frying time on total AC of hazelnut, corn, soybean and olive oils determined by DPPH method was observed by Karakaya and Şimşek (22–87, 18–87, 15–82, 16–75, 15–72 and 12–68 % for 0, 25, 50, 75, 100 and 125 min, respectively) [8].

The values obtained of RSD = 2.56–4.27 % indicate reasonable repeatability of the proposed DPPH method for the AC analysis of the unheated and heated rapeseed oils with different OLH.

### Correlations Between Thermal Stability and Antioxidant Capacity of Rapeseed Oils

Regression analysis was performed for the relationships among PV, *p*-AV, Totox, FFA, time to reach 25 % TPC and DPPH results of the studied rapeseed oils with various OLH. The calculated results of correlation coefficients (*r* = 0.8454–0.9919) indicated that there are significant, positive correlations between Totox values and PV, *p*-AV, FFA content in fresh and the heated oil samples (Table 2).

**Table 2:** Correlation coefficients (*r*) between parameters of thermal stability and antioxidant capacity of the unheated and heated rapeseed oils in the different layers height

	PV	*p*-AV	Totox	FFA	*t* (25 % TPC)
*p*-AV	0.7282				
Totox	0.8454*	0.9817**			
FFA	0.8017	0.9869**	0.9919**		
*t* (25 % TPC)	−0.1411	0.5248	0.3697	0.4231	
DPPH	−0.6930	−0.9980***	−0.9703*	−0.9776**	−0.5509

* *p* < 0.05; ** *p* < 0.001; *** *p* < 0.00001

This fact can be explained by FFA playing an important role in the oxidative stability of rapeseed oil, therefore they have relative contributions to the production of conjugated diene hydroperoxides and especially carbonyl compounds. However, significant, negative correlations for DPPH and *p*-AV, Totox values and FFA level was observed (*r* = −0.9703 to −0.9980). Thus, higher AC revealed rapeseed oils with lower amounts of secondary oxidation products and FFA. Although, DPPH values are insignificantly related to the PV (*r* = −0.6930, *p* = 0.1269) and time to achieve 25 % TPC (*r* = −0.5509, *p* = 0.2573) in the examined rapeseed oils. It is noteworthy that PV and time of creation 25 % TPC in the heated oils at different OLH did not correlate significantly with any determined parameters (Table 2). A lower insignificant correlation coefficient (*r* = 0.7282) between the PV and *p*-AV indicates that a high rate of hydroperoxide generation does not always involve a high rate of generation of secondary oxidation products.

## Conclusions

In this study, the results obtained suggest that, by increasing the OLH of the heated rapeseed oils, the SOS decreases significantly. With a reduction in SOS during pan frying, the rate of oil deterioration also decreases. Pan heating with the smallest OLH = 0.5 cm led to a noticeable deterioration of the frying oil. All chemical indicators of oil quality (PV, *p*-AV, Totox, FFA) had the highest undesirable values. Heating with a small OLH also led to the fastest time of TPC formation; a 0.5 cm layer reached 25 % TPC in a relatively short time (71.5 min), compared to the highest level 2.5 cm (315.1 min). Moreover the greatest decrease in the AC for the heated rapeseed oil with OLH = 0.5 cm was observed. The present study demonstrated the highly protective effect of increasing the OLH on the quality of the heated rapeseed oils.
